# Lipid Profiles of RAS Nanoclusters Regulate RAS Function

**DOI:** 10.3390/biom11101439

**Published:** 2021-09-30

**Authors:** Yong Zhou, John F. Hancock

**Affiliations:** Department of Integrative Biology and Pharmacology, University of Texas Health Science Center, Houston, TX 77030, USA; john.f.hancock@uth.tmc.edu

**Keywords:** RAS, nanoclusters, plasma membrane, phospholipids, phosphatidylserine, lipid acyl chain

## Abstract

The lipid-anchored RAS (Rat sarcoma) small GTPases (guanosine triphosphate hydrolases) are highly prevalent in human cancer. Traditional strategies of targeting the enzymatic activities of RAS have been shown to be difficult. Alternatively, RAS function and pathology are mostly restricted to nanoclusters on the plasma membrane (PM). Lipids are important structural components of these signaling platforms on the PM. However, how RAS nanoclusters selectively enrich distinct lipids in the PM, how different lipids contribute to RAS signaling and oncogenesis and whether the selective lipid sorting of RAS nanoclusters can be targeted have not been well-understood. Latest advances in quantitative super-resolution imaging and molecular dynamic simulations have allowed detailed characterization RAS/lipid interactions. In this review, we discuss the latest findings on the select lipid composition (with headgroup and acyl chain specificities) within RAS nanoclusters, the specific mechanisms for the select lipid sorting of RAS nanoclusters on the PM and how perturbing lipid compositions within RAS nanoclusters impacts RAS function and pathology. We also describe different strategies of manipulating lipid composition within RAS nanoclusters on the PM.

## 1. Introduction

The plasma membrane (PM) is the main functioning compartment for the highly oncogenic lipid-anchored RAS small GTPases [1,2,3,4,5,6]. The restrictive nature of RAS biological activities to the PM implies that RAS function and pathology can be very sensitive to changing PM properties. Of all the local environments in mammalian cells, the PM presents distinct conditions that are less appreciated and still poorly understood. Unlike previously thought, the PM is not a homogeneous fluid mixture. Rather, the PM contains nanometer-sized domains, each of which possesses distinct contents of proteins and lipids (with high specificities of headgroups and acyl chains) [7,8,9,10]. Because of the distinct proteolipid contents, these nano-domains display discrete fluidity, packing density, local curvature, mechanical (lateral elasticity and bending rigidity) and electrostatic properties, as well as various lifetimes of <1 s. Unlike the previous view that perturbing membranes globally affects all membrane contents in a universal fashion, perturbations may impact the mechanical and electrostatic properties of different PM nano-domains in distinct manners. Thus, perturbing the local membrane environment, on which RAS primarily replies for function and pathology, is now an exciting and novel strategy for interfering with RAS oncogenic activities.

Hydrophobic and electrostatic interactions between the isoform-specific C-terminal lipid-modified membrane-anchoring domains of RAS proteins and distinct lipids in the PM result in the formation of nanoclusters [11,12,13,14,15,16,17,18,19,20,21,22,23,24,25,26]. Consistently, ~50% of RAS molecules anchored to the PM exist as monomers, while the remaining RAS molecules on the PM are incorporated in nanoclusters [11,12,14]. On average, these RAS nanoclusters are ~20 nm in diameter, comprising 6–7 RAS molecules and possessing a lifetime of 100 ms to 1 s [11,12,14,27]. More specifically, RAS nanoclusters are heterogeneous in size and in the number of RAS molecules in each nanocluster. Typically, the numbers of RAS molecules in nanoclusters maintain a consistent population distribution: ~30% dimers, ~10% trimers and ~10% higher ordered oligomers [21,23]. Further, different RAS isoforms bound with either guanosine diphosphate (GDP) or GTP occupy different spaces on the PM, yielding spatially non-overlapping nanoclusters with distinct lipid and protein compositions [11,28,29]. RAS nanoclusters are biologically important for several reasons: (1) These nano-domains concentrate multiple RAS molecules and present larger targets for more efficient recruitment of effectors; (2) RAS nanoclusters also concentrate additional protein and lipid contents that are essential to the efficient binding of effectors (will be the focus of this article); (3) Nanoclusters provide a distinct environment to facilitate conformational orientation of RAS enzymatic G-domains for efficient binding of effectors; (4) RAS molecules within a nanocluster undergo dimerization and oligomerization for efficient effector binding; (5) Because RAS nanoclusters are signaling platforms for the GTP-bound active RAS to recruit effectors, the constitutively active mutants of RAS still require the formation of nanoclusters to efficiently recruit effectors and propagate signaling. As such, activities of the oncogenic mutant RAS can be manipulated via changing the structural integrity of nanoclusters of these mutant RAS. Taking together, RAS nanoclusters are biologically important for wild-type RAS and oncogenic mutants of RAS. In the current review, we will discuss in detail the structural constituents of RAS nanoclusters and how the structural integrity of these nano-domains can be modulated to manipulate RAS signaling.

### 1.1. RAS Nanoclusters Selectively Sort Distinct Lipids in the Plasma Membrane

**Figure 1:**
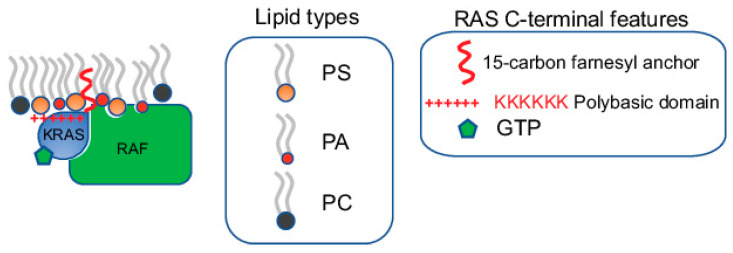
Specific lipids within RAS nanoclusters directly participate in effector recruitment. RAS nanoclusters possess distinct lipid profiles. The specific lipids enriched within these nanoclusters not only contribute to the structural integrity of the nanoclusters, but also directly participate in effector recruitment. Most RAS effectors contain specific lipid-binding domains. For example, RAF, a major KRAS effector, possesses separate PS- and PA-binding domains. Efficient recruitment of RAF to the PM, a key step in the activation of RAF, requires synergistic binding to both the GTP-bound active KRAS and specific lipids including PS and PA.

RAS nanoclusters comprise various other constituents, including actin cytoskeleton [12], galectin 1 [30,31,32,33], galectin 3 [34,35], ASPP2 [36], nucleophosmin and nucleolin [37,38], along with additional constituents yet to be discovered. Each component within these RAS nanoclusters plays important biological roles, which have been discussed in detail in various recent reviews [39,40,41]. Another set of major structural components of RAS nanoclusters is lipids, which are emerging targets of intense investigation. These lipids are biologically important because most RAS effectors, such as RAF (Rapidly accelerated fibrosarcoma) and phosphoinositol 3-kinase (PI3K), contain specific lipid-binding domains, in addition to RAS-binding domains. To be efficiently activated, these RAS effectors must synergistically bind to activated RAS molecules and distinct lipids in the PM. For example, CRAF (c-RAF), a major RAF isoform and a preferred effector of KRAS4B (Kirsten rat sarcoma splice variant 4B), possesses separate domains for binding phosphatidylserine (PS, in its cysteine-rich domain) and phosphatidic acid (PA, in its C-terminus) [42,43,44,45] (Figure 1). The Binding of PS and PA, in addition to binding of the GTP-bound active RAS, is required for the proper activation and the kinase activity of CRAF [42,44] (Figure 1). PI3K is a preferred effector of HRAS (Harvey rat sarcoma) [46,47,48] and converts phosphoinositol 4,5-bisphosphate (PIP2) to phosphoinositol 3,4,5-trisphosphate (PIP3). As such, concentrating PS and PA within KRAS4B nanoclusters, or pre-assembling PIP2 molecules in HRAS nanoclusters, is important to the biological function of RAS isoforms. Since the constitutively active RAS still requires the same sets of lipids nearby to properly recruit effectors, the pathological activities of RAS still depend on how effective they can form nanoclusters. As such, perturbation of RAS nanoclusters not only disrupts the concentration of multiple RAS molecules, but also alters the precise lipid composition within these nano-domains, both of which compromise effector recruitment and signal transduction. Taking together, altering the lipid composition of RAS nanoclusters is an encouraging novel strategy for interfering with RAS pathology. Here, we will discuss the latest findings on the precise lipid profiles of RAS nanoclusters, molecular mechanisms of selective lipid sorting by different RAS isoforms. We will also summarize how RAS isoforms respond to changing membrane properties in distinct manners, as well as the intriguing specificity of how changing lipid profiles of the PM may impact the spatiotemporal organization and function of different RAS isoforms in distinct manners.

### 1.2. Electron Microscopy-Spatial Analysis Quantifies the Nanoclustering and Selective Lipid Sorting of RAS on Intact Plasma Membrane

Lipid profiles of RAS nanoclusters have been studied via quantitative super-resolution imaging, in vitro biophysical assays and in silico molecular dynamic (MD) simulations in a wide variety of model systems including intact tissues, cultured mammalian cells, synthetic bilayers and simulated membranes. Specifically, electron microscopy (EM)-spatial analysis, including univariate nanoclustering analysis and bivariate co-clustering analysis, is a quantitative super-resolution imaging method and has been particularly instrumental in characterizing the spatiotemporal organization of RAS on the PM [11,12,21,22,23,24,25]. The spatial parameters determined via the EM-spatial analysis include the extent of lateral nanoclustering, PM localization, monomers/dimers/oligomers population distribution and optimal radii (in nanometers) of nanoclusters of RAS proteins, as well as the selective enrichment of distinct lipids, actin and other proteins in RAS nanoclusters. The protocols of the EM-spatial analysis have been described in detail recently in several reviews [49,50]. Briefly, to calculate effects of different lipids on the extent of nanoclustering of RAS, the GFP-tagged RAS molecules anchored to the inner leaflet of the PM are immunolabeled with 4.5 gold nanoparticles conjugated to anti-GFP antibody. Transmission EM (TEM) is used to image the gold distribution on intact PM sheets attached the EM grids. The statistical Ripley’s K-function analysis calculates the extent of nanoclustering of the gold nanoparticles. To quantify the lipid composition of RAS nanoclusters, a bivariate EM co-clustering analysis calculates the extent of co-localization between a GFP-tagged specific lipid-binding domain and an RFP-tagged RAS on intact PM sheets. GFP and RFP on intact PM sheets are immunolabeled with 2 nm gold bound to anti-GFP and 6 nm gold-anti-RFP, respectively. Co-clustering between the two populations of gold particles is calculated using a Ripley’s bivariate co-clustering analysis. A statistically meaningful co-clustering between a lipid-binding domain and RAS suggests that the specific lipid type probed by the lipid-binding domain is enriched within RAS nanoclusters studied. The enrichment of a lipid type within RAS nanoclusters is validated via various depletion and subsequent acute addback protocols to carefully examine in parallel how acute supplementation of different natural extracts and synthetic exogenous phospholipids may restore/impact the nanoclustering and PM localization of RAS in distinct manners. Findings using the EM-spatial analysis have been mostly corroborated in quantitative fluorescence imaging methods in intact/live cells and intact tissues, including fluorescence lifetime imaging microscopy-fluorescence resonance energy transfer (FLIM-FRET), fluorescence recovery after photobleaching (FRAP), total internal reflection fluorescence-single particle tracking (TIRF-SPT), Raster image correlation spectroscopy (RICS) and photoactivable localization microscopy (PALM). Nanoclustering of RAS, as well as some of their lipid environment, has also been consistently observed in vitro, including supported bilayers via atomic force microscopy (AFM), spherical synthetic vesicles and isolated PM blebs via FLIM-FRET and molecular dynamics (MD) simulations [15,16,17,19,24,51]. The use of these powerful quantitative imaging methods in model systems with varying complexities allows the detailed characterization of the lipid profiles in the nanoclusters of different RAS isoforms.

## 2. KRAS4B Nanoclusters Selectively Enrich Lipids with Distinct Headgroup and Acyl Chain Structures

### 2.1. KRAS4B Nanoclusters on the PM Contain Distinct and Precise Molecular Contents Important for Signal Propagation

KRAS4B (hereafter simply referred to as KRAS) is the most prevalent RAS isoform in cancer, with its oncogenic mutants found in ~80% of all RAS-dependent tumors, especially in 98% of pancreatic, 45% of colorectal and 31% of lung tumors [1,2,3,6]. KRAS remains one of the most difficult targets to pharmaceutically inhibit, most likely because the dynamic and globular G-domain of KRAS has been difficult for inhibitors to bind. Interestingly, KRAS function and pathology are mostly compartmentalized to the plasma membrane (PM) [1], where KRAS uses its C-terminal membrane-anchoring domain to interact with a distinct set of lipids. More specifically, two important features of the membrane-anchoring domain of KRAS are required: a hexa-lysine polybasic domain (PBD, amino acids 175–180) and a poly-unsaturated and branched 15-carbon farnesyl chain covalently linked to its C-terminal Cysteine (Cys) 185 [52,53]. Early fractionation assays and fluorescence imaging identified that the absence of either feature significantly compromised the ability of KRAS to localization to membranes [52,53]. Later EM-nanoclustering analysis showed that the GFP-tagged inactive KRAS-GDP, active KRAS-GTP, the truncated C-terminal hypervariable region CTK (amino acids 167–185) or the minimal anchoring domain tK (amino acids 175–185) effectively formed nanoclusters on the PM [11,12,21]. In addition of 6–7 KRAS molecules (on average), each KRAS nanocluster also comprises other proteins and lipids with a precise composition. The past several decades of intense studies have revealed a complex protein content within KRAS nanoclusters on the PM. In particular, disruption of actin cytoskeleton, via the treatment of Latrunculin A, significantly disrupted the nanoclustering of GFP-KRAS on the PM [12,21,24]. This suggests that KRAS nanoclusters are dependent on actin. EM-nanoclustering, in combination of FLIM-FRET and other imaging techniques, further discovered other important protein components of KRAS nanoclusters, including galectin-3 (Gal-3), nucelophosmin and nucleolin [34,35,37,38]. Depletion of any of these components significantly compromised the nanoclustering of KRAS, and in turn inhibited the signal output of the KRAS-dependent mitogen-activated protein kinases (MAPKs) signaling cascades. Thus, KRAS nanoclusters comprise various actin and protein constituents crucial to the structural integrity of these nano-domains and consequentially directly and indirectly participate in the effector recruitment and signal propagation of KRAS nanoclusters. Future studies may discover additional proteins in KRAS nanoclusters.

### 2.2. KRAS Nanoclusters Contain Distinct Lipid Types with Specific Headgroups, with a Selective Enrichment of PS Lipids

**Figure 2:**
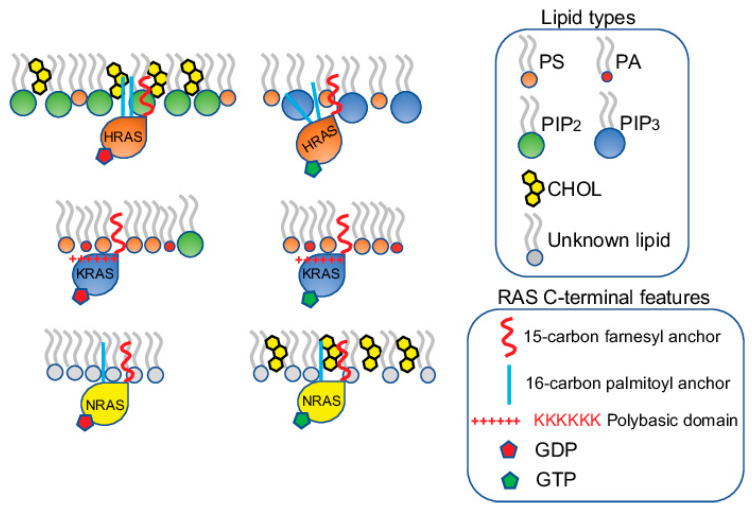
RAS proteins form spatially distinct nanoclusters in isoform- and guanine nucleotide-specific manners. HRAS, KRAS and NRAS each form non-overlapping nanoclusters on the PM. For each isoform, the GDP-bound inactive and the GTP-bound active forms also form separate nanoclusters. The nanoclusters of the inactive GDP-HRAS contain PIP2 and cholesterol, while the active GTP-bound HRAS selectively associates with PIP3. The active and inactive KRAS contain similar lipid contents, enriching PS and PA. The nanoclusters of the inactive KRAS contain additional PIP2. The lipid environments of the inactive and active NRAS are less clear, except that cholesterol is more enriched in the nanoclusters of the active GTP-bound NRAS.

EM-bivariate co-clustering analysis showed that the RFP-tagged full length constitutively active mutant KRASG12V or the truncated minimal membrane-anchoring domain tK co-clustered with lipid-binding domains GFP-LactC2 specifically probing PS and GFP-PASS tagging PA, but not GFP-PH-PLCδ with specific affinity for PIP2, GFP-PH-Akt for PIP3 and GFP-D4H for cholesterol [21,22,23]. These data suggest that KRAS nanoclusters selectively enrich PS and PA, but not PIP2, PIP3 and cholesterol (Figure 2). This is consistent with the notion that CRAF, a preferred effector of KRAS, possesses specific PS- and PA-binding domains [42,44]. Indeed, acute cholesterol depletion did not affect the nanoclustering of GFP-KRAS, including the GDP-/GTP-bound full-length, the truncated C-terminal hypervariable region CTK or the minimal membrane-anchoring domain tK, consistent with the notion that KRAS nanoclusters are cholesterol independent [11,25]. Atomic force microscopy (AFM) showed that the purified full-length KRAS localized to the cholesterol-poor liquid-disordered (Ld) domains in supported bilayers [16]. The similar affinity of KRAS anchor for the cholesterol-poor Ld has also been predicted in coarse-grained MD simulations [17]. The PS enrichment is interesting because PS is an abundant anionic phospholipid primarily concentrated in the inner leaflet of the PM, comprising ~20–30% of all lipid contents in the PM inner leaflet. In EM-nanoclustering analysis, depleting the endogenous PS, via knocking down a PS synthase PSS1 [54], in PSA3 cells (a mutant Chinese hamster ovarian cell line) mislocalized GFP-KRAS from the PM and disrupted the nanoclustering of KRAS molecules left on the PM [21,22,23,24,25]. Further EM-univariate nanoclustering analysis showed that increasing the endogenous PS levels, via supplementation of different concentrations of ethanolamine (Etn) to the PS-depleted PSA3 cells, dose-dependently restored the PM localization and the nanoclustering of GFP-KRAS on the PM [21]. In the PS-depleted PSA3 cells, acute addback of exogenous PS extracted from mouse brain also effectively restored the nanoclustering of GFP-KRAS [21,22,23,24,25,55]. On the other hand, acute addback of mouse brain extracts of PIP2, PC, PE or cholesterol did not impact the nanoclustering of GFP-KRAS in the PS-depleted PSA3 cells [55]. These data strongly suggest that KRAS nanoclusters selectively enrich PS. Further functional assays showed that depleting endogenous PS (via PSS1 knockdown) or mislocalizing PS from the PM (via treatment of fendiline to inhibit acid sphingomyelinase) effectively compromised the KRAS-regulated phosphorylation of RAF/MEK/ERK in the MAPK cascade and the proliferation of the mutant KRAS-dependent human tumor lines, without affecting the KRAS-independent human tumor lines. Similar sensitivity of KRAS mutant activities to PS was also found in vivo in xenografts of human tumor lines [55,56,57,58]. These data further reenforce the selective dependence of KRAS nanoclustering, signaling and function on PS lipids in vitro, in cultured cells and in vivo.

### 2.3. KRAS Nanoclusters Selectively Sort Distinct PS Species with Specific Acyl Chain Structures

A key feature facilitating the lipid interactions of KRAS is its C-terminal membrane-anchoring domain comprising 10 positively charged lysine residues, especially its contiguous hexa-lysine polybasic backbone (Lys175–180), suggesting a strong electrostatic interaction between KRAS and the PM. The specific enrichment of PS, which is the most abundant anionic phospholipid in the PM inner leaflet and a major contributor to the negative charges on the cytosolic surface of the PM, further reenforces the electrostatic nature of the selective lipid sorting of KRAS. This is indeed supported by in vitro surface plasmon resonance (SPR) binding assays, where the binding of the purified KRAS to zwitterionic bilayers without anionic lipids was very weak [59]. Interestingly, KRAS nanoclustering and activities depended on the monovalent PS, but not the more highly charged PIP2 or PIP3 [21,22,23,24], suggesting more than just electrostatics. The non-electrostatic contribution to the KRAS lipid sorting capability suggests extensive interactions between the KRAS anchor and the bilayer core, hence potential sensitivity for the packing density of lipids and the hydrophobic interactions. This is supported by evidence that KRAS nanoclusters possess ability to sort lipid acyl chains, the packing of which contributes to packing density and hydrophobic properties of membranes. Specifically, in PSA3 cells depleted of endogenous PS, different synthetic PS species were acutely added back [23,24,25]. These PS species included the fully saturated di18:0 PS (DSPS), mono-unsaturated di18:1 PS (DOPS), di-unsaturated di18:2 PS (DLPS), the mixed-chain 16:0/18:1 PS (POPS) and the mixed-chain 18:0/18:1 PS (SOPS) [23,24,25]. EM analysis of intact PM sheets, fluorescence imaging and lipidomics consistently showed these different PS species equivalently incorporated into cells and properly transported to the inner leaflet of the PM [23,24,25]. With the identical phosphoserine headgroup, these PS species should interact with KRAS electrostatically in the same fashion. The differences arise from their distinct acyl chains, thus giving rise to distinct packing patterns and mechanical properties. This acyl chain-dependent membrane mechanics has been extensively characterized in phosphatidylcholine (PC) species with acyl chains of different lengths and unsaturation levels [7,8,9,10]. PS species with distinct acyl chains also spatially segregate to distinct regions of the PM. This is reflected by the EM-bivariate co-clustering analysis, where the PS-binding domain GFP-LactC2 co-localized extensively with the cholesterol-binding domain RFP-D4H in the PS-depleted PSA3 cells supplemented with the fully saturated DSPS, but not when supplemented with the unsaturated DOPS or the mixed-chain POPS [25]. This data suggests that the fully saturated DSPS, but not other PS species tested, exhibit strong cholesterol affinity and different PS species distribute to spatially distinct regions of the PM. Further EM-nanoclustering analysis showed that the acute addback of the fully saturated DSPS had no effect on the spatiotemporal organization (including PM localization and lateral nanoclustering) of GFP-KRASG12V, while the unsaturated PS species (DOPS and DLPS) and the mixed-chain PS species (POPS and SOPS) effectively restored the localization of GFP-KRASG12V to the PM [23,25]. Intriguingly, only the mixed-chain POPS and SOPS effectively restored the lateral nanoclustering of GFP-KRASG12V on the PM of PSA3 cells depleted of endogenous PS [23,25]. EM-bivariate co-clustering analysis further illustrated that the recruitment of effector CRAF to KRAS nanoclusters only occurred when the PS-depleted PSA3 cells were supplemented with the mixed-chain POPS, but not other synthetic PS species examined [23]. These data suggest the sensitivity of the spatial distribution of KRAS to PS acyl chain composition. Further, EM-bivariate co-clustering analysis showed that the RFP-tagged KRASG12V with the original hexa-lysine co-localized with the PS-binding domain GFP-LactC2 in PSA3 cells depleted of endogenous PS and acutely supplemented with the mixed-chain POPS and SOPS, but not other PS species tested [23,25]. Taken together, KRAS nanoclusters selectively enrich mixed-chain PS species, thus possessing distinct abilities to sense and respond to changing the lipid acyl chain-dependent membrane properties.

## 3. Conformational Sampling of Polybasic Domain Contributes to Selective Lipid Sorting of KRAS

It is intriguing that KRAS polybasic domain enriched with many charged residues (10 lysines within the last 17 residues of its C-terminal membrane-anchoring domain) interacts with membranes non-electrostatically. Polybasic domains have been traditionally thought to adopt more random structures. However, an earlier MD simulation predicted that KRAS anchor preferred a pseudo-helical hairpin structure on a negatively charged bilayer [60]. Further EM-spatial analysis examined single point mutants of the KRAS polybasic domain, K175Q, K176Q, K177Q, K178Q, K179Q and K180Q [23]. These polybasic domain mutants share identical charges, but sort distinct lipids. In particular, a series of EM-bivariate co-clustering analyses compared in parallel the co-localization between the GFP-tagged lipid-binding domains and the RFP-tagged KRASG12V with the original polybasic domain or single-point mutations [23]. More strikingly, when compared with the nanoclusters of KRAS with the original hexa-lysine polybasic domain, the nanoclusters of KRAS.K177Q and KRAS.K178Q more strongly interacted with PIP2 and no longer enriched PS [23]. These quantitative imaging analyses suggest that specific residues within KRAS polybasic domain possess precise coding for select lipid sorting. This is corroborated by all-atom MD simulations predicting that the KRAS polybasic domain mutants sampled among various well-defined conformational orientations, including the ordered (O), intermediate (I) and disordered (D) states [23]. More specifically, the original KRAS polybasic domain favored the D states, with ~64% of the simulated polybasic anchors adopting the D states, 29% sampling the I states and 6% sampling the O states [23,25]. The mutant K177Q or K178Q anchors, on the other hand, switched their conformational sampling to favor the O states, with 42% of K177Q and 25% of K178Q mutant anchors (as opposed to 6% of the original KRAS anchor) sampling the O states [23,25]. Further all-atom MD simulations predictedthat the KRAS anchor in the D states associated more extensively with the PS headgroup than the anchors adopting the O and I states [23], providing a molecular mechanism for how KRAS selectively sorts PS.

**Figure 3:**
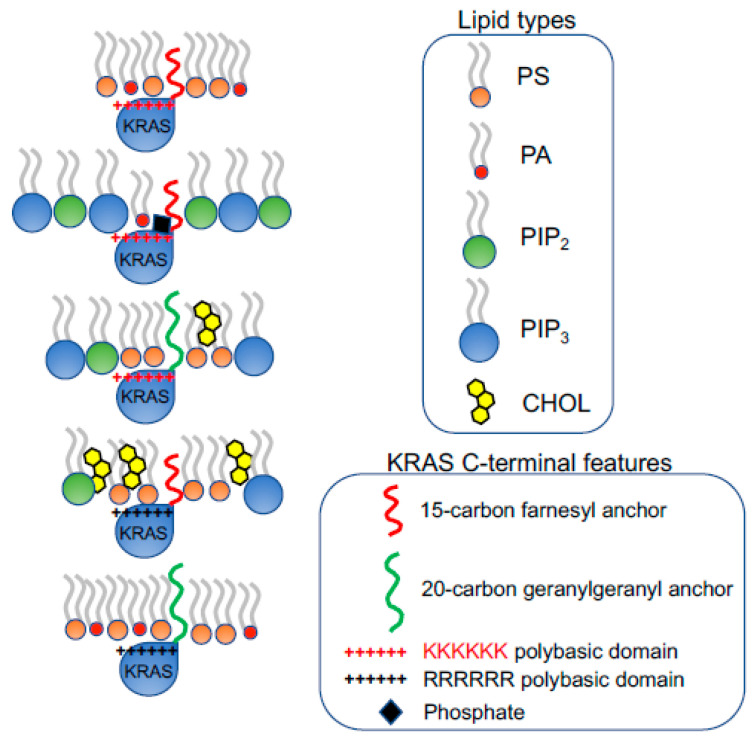
KRAS polybasic domain contributes to the selective sorting of lipid headgroups. The prenylation (15-carbon farnesyl or 20-carbon geranylgeranyl chain) and the polybasic domain of KRAS combine to contribute to the selectivity of lipid headgroups within KRAS nanoclusters. The constitutively active oncogenic mutant KRASG12V with different combinations of the prenyl anchors and the residues of polybasic domains selectively sort distinct lipid types with different headgroups.

**Figure 4:**
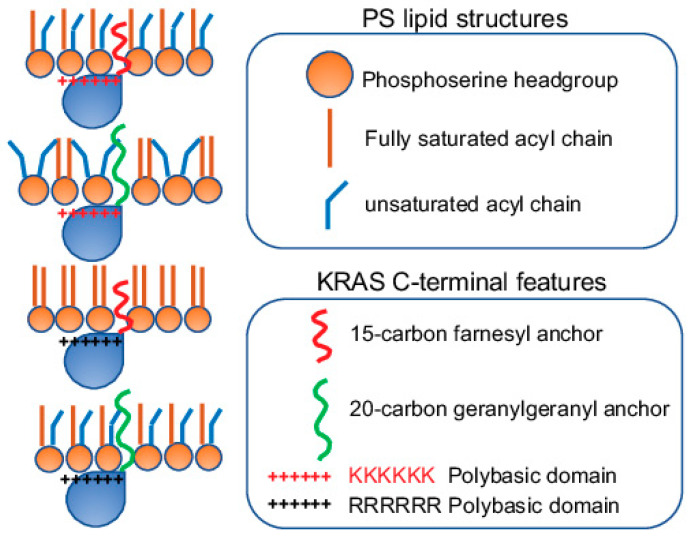
The prenylated polybasic domain of KRASG12V possesses capability to selectively sort PS acyl chains. Despite highly charged, the prenylated polybasic domain of KRASG12V distinguishes the acyl chain structures of PS lipids. In particular, four KRASG12V constructs with equivalently charges but distinct prenyl anchors and/or basic residues within the polybasic domain sort different PS species with distinct acyl chains. This selective sorting of lipid acyl chains contributes to the distinct abilities of KRASG12V polybasic domain mutants to respond to different membrane properties, including transmembrane voltages, membrane curvature, cholesterol depletion, etc.

The conformational sampling of the equivalently charged KRAS polybasic domain anchor constructs, including the original farnesylated hexa-lysine anchor, farnesylated hexa-arginine polybasic domain (denoted as 6R), geranylgeranylated hexa-lysine polybasic domain (denoted as C20), and a geranylgeranylated hexa-arginine polybasic domain (denoted as 6R-C20), also differs significantly. These 4 KRAS polybasic domain constructs contain identical number of charged residues and undergo similar electrostatic interactions with the charged membranes. Interestingly, the geranylgeranylated C20 and the hexa-arginine 6R KRAS anchors favored the D states, with the 6R anchor adopting exclusively the D states [23,25]. The combo mutant 6R-C20 KRAS anchor adopted similar distribution of D, I and O states as the KRAS anchor with the original polybasic domain [23,25]. The distinct conformational sampling correlates with the selective lipid sorting of these equivalently charged KRAS polybasic domain mutants (Figure 3). EM-bivariate co-clustering analysis between the GFP-tagged lipid-binding domains and the RFP-tagged KRASG12V with the original or the equivalently charged mutant polybasic domains showed that these mutants with equivalent charges sort distinct lipids types. As described above, the nanoclusters of GFP-KRASG12V with the original polybasic domain selectively sorted PS and PA [23]. The nanoclusters of GFP-KRASG12V.6R more favorably sorted cholesterol but less with PA when compared with GFP-KRASG12V with the original farnesylated hexa-lysine polybasic domain [23]. On the other hand, GFP-KRASG12V.C20 more extensively interacted with PIP2 and PIP3 [23]. GFP-KRASG12V.6R-C20 sorted similar lipid types as GFP-KRASG12V with the original polybasic domain [23]. These equivalently charged KRAS polybasic domain constructs also associate with distinct PS species with different acyl chain structures (Figure 4). As described above, in the synthetic PS acute addback experiments using PSA3 cells depleted of endogenous PS, EM-bivariate co-clustering analyses showed that GFP-KRASG12V with the original farnesylated hexa-lysine polybasic domain selectively sorted the mixed-chain POPS but not other PS species tested [25]. The nanoclusters of GFP-KRASG12V.6R selectively enriched the fully saturated DSPS and no longer associated with POPS, while the nanoclusters of GFP-KRASG12V.C20 enriched DSPS and the mono-unsaturated DOPS and no longer associated with POPS [25]. Nanoclusters of GFP-KRASG12V.6R-C20 behaved similar as the original KRAS, selectively sorting the mixed-chain POPS, but not other PS species tested [25]. These parallel comparisons nicely illustrate that electrostatics and non-electrostatics contribute to the selective lipid sorting of KRAS on the PM, and that individual residues encode intricate capabilities to selectively sort lipid headgroups.

## 4. Selective Lipid Sorting Capability Mediates Distinct Responses of KRAS Nanoclustering and Signaling to Perturbations of Membrane Properties

### 4.1. PM Depolarization Enhances the Nanoclustering and Signaling of Oncogenic Mutant KRAS

Extensive biophysical studies establish tight correlations among lipid structures, the spatial distribution of lipids in bilayers and membrane properties [61]. The ability of KRAS to selectively sort distinct lipid headgroups and acyl chains suggests that KRAS nanoclusters on the PM may act as transducers to allow cells to convert between membrane perturbations and the KRAS-regulated intracellular signaling cascades. An intriguing example is the connection between the transmembrane voltage and tumor development. In particular, the PM of tumor cells has long been characterized to be more depolarized than their normal counterparts [62,63,64,65]. Since concentration gradient of potassium ions across the PM is mainly responsible for establishing the transmembrane potential, a series of potassium ion channels has been shown to participate in cell growth, proliferation and apoptosis [62,63,64,65]. However, how intracellular mitogenic signaling respond to changing surface electric potential is still less clear. EM-univariate nanoclustering analysis revealed that the nanoclustering of the GFP-tagged full-length oncogenic mutant KRASG12V, as well as the minimal membrane-anchoring domain tK, elevated upon PM depolarization in dose- and time-dependent manners [22]. On the other hand, the nanoclustering of the GFP-tagged HRAS, or its minimal membrane-anchoring domain tH, was completely insensitive to changing transmembrane voltages [22]. Concordantly, the MAPK signal output of the oncogenic mutant KRASG12V-transformed mammalian cells, as well as intact *Drosophila* embryo expressing a KRAS ortholog, elevated upon PM depolarization [22]. PM depolarization also promoted the nanoclustering of PS and induced further enrichment of PS within KRAS nanoclusters [22,25]. Depletion of the endogenous PS effectively abolished the responses of KRAS nanoclustering and signaling to changing transmembrane voltages. Supplementation of Etn, which effectively recovered the endogenous PS level in PSA3 cells depleted of endogenous PS, restored the sensitivity of KRAS nanoclustering and signaling to PM depolarization [22,25]. Intriguingly, when comparing the effectiveness of specific PS species acutely added back to PSA3 cells depleted of the endogenous PS, only the mixed-chain POPS, but not the symmetric species DSPS and DOPS, effectively restored the sensitivity of GFP-KRASG12V to PM depolarization [25]. The non-electrostatic contribution of responses of KRAS to changing PM electric voltages is further reflected in a set of EM-univariate nanoclustering analyses comparing in parallel 4 KRAS polybasic domain mutants with equivalent numbers of charged residues [25]. In particular, PM depolarization effectively further enhanced the nanoclustering of KRAS with the original farnesylated hexa-lysine polybasic domain, KRAS with a geranylgeranylated hexa-lysine polybasic domain and KRAS with a geranylgeranylated hexa-arginine polybasic domain [25]. On the other hand, depolarizing the PM had no effect on the nanoclustering of KRAS with a farnesylated hexa-arginine polybasic domain [25]. Taken together, PM depolarization selectively promotes the nanoclustering of KRAS, but not that of HRAS, on the PM. PS mediates the responses of KRAS nanoclustering and signaling to changing transmembrane voltages.

### 4.2. PM Curvature Disrupts the Nanoclustering and Signaling of KRAS

**Figure 5:**
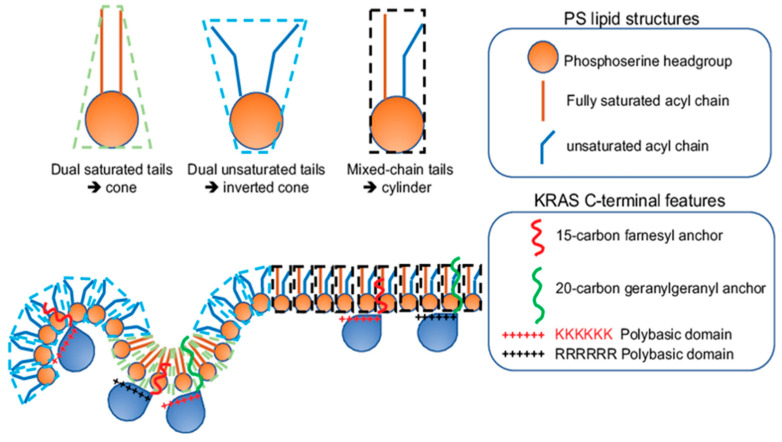
KRAS polybasic domain mutants with equivalent charges possess distinct preferences for membrane curvature. KRASG12V with the original farnesylated hexa-lysine or the geranylgeranylated hexa-arginine prefers to form nanoclusters on flatter membranes with low curvature. On the other hand, KRASG12V with the farnesylated hexa-arginine or the geranylgeranylated hexa-lysine polybasic domain favors to interact with more curved membranes. The ability of these KRAS polybasic domain mutants to selectively sort distinct PS species with different packing geometries contributes to the distinct membrane curvature sensing capabilities of KRAS.

Cell surface curvature defines cell morphology, which has been shown to correlate with biological functions including growth, proliferation and apoptosis [66,67,68]. Specifically, micropatterning and microplating technologies have been used extensively to confine mammalian cells to various shapes and cell functions have been examined [68]. Consistently, cells with flat and round epithelial morphology undergo more growth and proliferation and less apoptosis than the same cells confined to more elongated fibroblast-like morphologies [68]. Since RAS proteins are important upstream regulators of cell growth, proliferation and apoptosis and functions of RAS proteins are mostly compartmentalized to the cell surface, RAS nanoclusters on the PM may be important mediators for how intracellular signaling communicates with cell morphology. A recent study showed that the nanoclustering of RAS proteins responded to changing membrane curvature in an isoform-specific manner. Specifically, consistent in EM-univariate nanoclustering analyses on intact PM sheets, Raster image correlation spectroscopy in live cells, whole cells grown on arrays of nano-fabricated nanobars, fluorescence lifetime imaging combined with fluorescence resonance energy transfer in isolated PM blebs and synthetic two-component vesicles, induction of positive curvature of the PM (curving toward the cytosol) compromised the PM localization and nanoclustering of the full-length oncogenic mutant KRASG12V or the minimal membrane anchor tK [24]. Additionally, consistently in EM-univariate nanoclustering analyses on intact PM sheets and fluorescence lifetime imaging combined with fluorescence resonance energy transfer in isolated PM blebs, higher positive curvature of the PM further elevated the nanoclustering and PM localization of the full-length oncogenic mutant HRASG12V or its minimal membrane anchor tH [24]. Intriguingly, induction of negative PM curvature (curving away from the cytosol), via expression of a negative curvature-inducing inverse BAR domain from IRSp53, did not impact the nanoclustering and PM localization of KRAS, but significantly disrupting those of the HRAS anchor [24]. Interestingly, when the endogenous PS was depleted in PSA3 cells, the spatiotemporal organization of KRAS no longer responded to changing PM curvature, which was recovered by the acute addback of the mixed-chain POPS but not other PS species tested [24]. Concordantly, the binding of the purified and the fully processed KRAS favored larger and flatter vesicles containing 20% POPS, but became independent of size of vesicles containing DOPS [24]. Further evidence that the membrane curvature sensing of KRAS is mediated by its ability to selectively sort lipids came from a parallel comparison among 4 equivalently charged KRAS polybasic domain constructs (Figure 5). Specifically, elevating PM curvature disrupted the nanoclustering and reduced PM localization of GFP-KRASG12V with the original polybasic domain and GFP-KRASG12V.6R-C20, both of which selectively sort the mixed-chain POPS [25]. On the other hand, the nanoclustering of GFP-KRASG12V.6R and GFP-KRASG12V.C20, both of which prefer to associate with the fully saturated DSPS and/or the mono-unsaturated DOPS, favors the more curved PM [25]. This is nicely consistent with the EM-univariate nanoclustering analysis showing that elevating the PM curvature disrupted the spatial segregation of POPS, but further enhanced the spatial segregation of DSPS and DOPS [24] (Figure 5). Thus, experiments in both synthetic vesicles and intact PM sheets consistently show that the membrane curvature sensing of KRAS is specifically mediated by distinct PS species. MAPK signaling in the RAS-less mouse embryonic fibroblast (MEF) line (in the absence of all endogenous RAS isoforms) expressing only oncogenic mutant KRASG12V was further promoted upon hypotonicity-induced flattening of the cell PM [24]. MAPK signaling in the RAS-less MEF line (no endogenous RAS) expressing BRAFV600E, a constitutively active oncogenic mutant of a major KRAS effector, did not respond to hypotonic flattening [24]. These data consistently suggest that nanoclustering and signaling of KRAS favor flatter PM with low curvature. The mixed-chain PS species selectively mediate the membrane curvature sensing capability of KRAS.

### 4.3. KRAS Polybasic Domain Mutants Possess Distinct Dependence on Cholesterol in the PM

Cholesterol is an important part of biomembranes and a major driver for phase separation of model bilayers and compartmentalization of biological membranes. In mammalian cells (through treatment of methyl β-cyclodextrin, MβCD, to deplete cellular cholesterol), supported bilayers and molecular dynamic simulations, RAS isoforms, such as HRAS and NRAS, have been shown to depend on cholesterol levels for their nanoclustering [11,13,15,17,19,69]. Interestingly, EM-spatial analyses consistently show that the PM localization and the nanoclustering of KRAS were independent of acute cholesterol depletion by MβCD treatment [11,16,25]. This is consistent among the full-length wild-type GDP-/GTP-bound KRAS, the constitutively active mutant KRASG12V or the truncated minimal anchoring domain tK. The cholesterol independence of KRAS was further supported in synthetic supported bilayers and coarse-grained MD simulations, illustrating that the tK anchor preferentially partitioned to the cholesterol-poor liquid-disordered, Ld, domains [16,17,59,60]. Interestingly, recent EM-nanoclustering analysis showed that acute cholesterol depletion via MβCD effectively disrupted the nanoclustering of GFP-KRASG12V.C20 and GFP-KRASG12V.6R [25]. This is consistent with the notion that both GFP-KRASG12V.C20 and GFP-KRASG12V.6R preferentially sort the fully saturated DSPS, which significantly co-localized with cholesterol in EM-bivariate analysis [25]. Taking together, the sequence of the polybasic domain and the structures of the prenyl anchor together determine the cholesterol affinity of KRAS.

## 5. Strategies of Therapeutically Perturbing Lipid Profiles of RAS Nanoclusters

**Figure 6:**
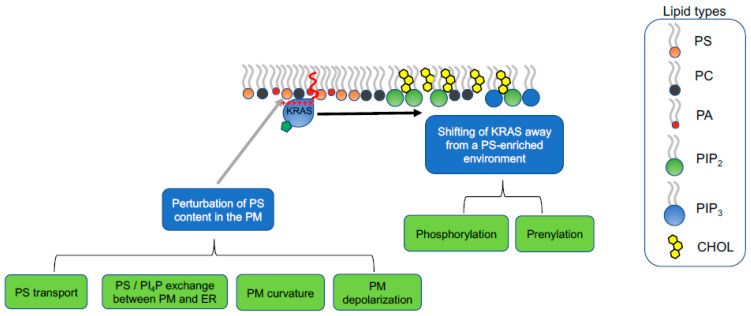
The select lipid sorting of KRAS can be targeted to manipulate KRAS function and pathology. The nanoclusters of the constitutively active oncogenic mutant KRAS enrich PS lipids. Two different strategies can be used to compromise KRAS nanoclustering, thus effector recruitment and signal transduction: (1) perturbation of the PS content in the plasma membrane; (2) alteration of KRAS polybasic domain to change the lipid preferences of KRAS.

Because KRAS nanoclusters selectively enrich PS lipids, perturbing the PS contents in the PM may present an opportunity to attenuate KRAS oncogenesis. There can be three venues to perturb the PS enrichment in KRAS nanoclusters in the PM: (1) changing PS metabolism to decrease the total PS levels; (2) alteration of PS intracellular transport to disrupt the extent of PS trafficked to the PM; (3) shifting the conformational sampling of the C-terminal polybasic domain of KRAS to modulate the select lipid sorting by KRAS (Figure 6). PS is mostly converted from phosphatidylcholine (PC) by PS synthase 1 (PSS1) and phosphatidylethanolamine (PE) by PSS2, via headgroup exchange on the ER membranes [54]. As described above, knocking down PSS1 in CHO cells effectively mislocalized KRAS from the PM and disrupted the nanoclustering of KRAS molecules left on the PM [21,22,23,24,25]. The disrupted spatial distribution of KRAS on the PM was efficiently restored by supplementation of ethanolamine (Etn), which is an upstream ligand in the PE biosynthesis, stimulates PSS2 activities and restores endogenous PS levels [21,22,23,24,25]. In CHO cells with PSS1 knocked out, acute supplementation of synthetic mixed-chain PS species (POPS and SOPS), but not other PS species, effectively recovered the nanoclustering of KRAS on the PM [23,25]. Concordantly, knocking down PSS1 inhibited the KRAS-dependent MAPK signaling, which was restored by Etn supplementation [22]. Thus, perturbing the total endogenous PS levels in cells effectively disrupts the nanoclustering of KRAS and abolishes KRAS signaling.

### 5.1. Interfering with PS Trafficking through Recycling Endosomes

Once synthesized on the ER membrane, PS is actively transported through various endomembrane compartments for eventual localization to the PM inner leaflet [54]. A major endomembrane compartment for mediating PS transport is the recycling endosomes, which are main reservoirs of PS and secret transport vesicles containing PS lipids to the PM [55,58,70,71,72]. Another route for PS transport is an exchange between PS in the ER and phosphoinositol 4-monophosphate (PI4P) in the PM [73,74,75,76]. We will discuss in detail how PS transport in cells can be modulated pharmacologically to manipulate biological activities of oncogenic mutant KRAS.A key contributor to the proper trafficking of various lipids, including PS and cholesterol, among the PM and the endomembranes is a homeostasis between sphingomyelin (SM) and ceramide (Cer) [77,78,79]. Fendiline, an inhibitor of the voltage-gated L-type calcium channels, has been found to effectively inhibit the activities of acid sphingomyelinase (ASM), which hydrolyzes SM to Cer [55,58,77]. Confocal imaging showed that, in mammalian cells treated with fendiline (<15 μM for 24–48 h), labeling of the GFP-tagged SM-specific-binding domain, lysenin, was markedly elevated on the PM outer leaflet (the main compartment for SM), as well as intracellularly [55,58]. Lipidomics further showed that fendiline treatment significantly reduced Cer levels by 20%, consistent with the fendiline-altered SM-Cer homeostasis [55]. As a result, PS and cholesterol were mislocalized from the PM of mammalian cells treated by fendiline at concentrations well below its channel-inhibiting doses [55,58]. In further EM-nanoclustering and confocal imaging experiments, the fendiline treatment consequentially led to mislocalization of the GFP-tagged oncogenic mutant KRASG12V from the PM, disruption of the nanoclustering of GFP-KRASG12V left on the PM [55,57,58]. This is concordant with the fendiline-led inhibition of the KRASG12V-dependent MAPK signaling, compromised the proliferation the mutant KRAS-transformed cancer cells, as well as decreased the sizes of mutant KRAS-dependent tumors in xenograft models [55,57,58]. Thus, compromising PS intracellular trafficking via fendiline treatments effectively attenuates the spatiotemporal organization and biological function of oncogenic mutant KRAS.

### 5.2. Perturbation of PI4P/PS Exchange at the PM/ER Contact Sites

Exchange of PS between the ER and the PM inner leaflet is facilitated by oxysterol-binding protein (OSBP)-related binding proteins (ORPs), including ORP5 and ORP8, located at the PM/ER contact sites [73,74,75,76] (Figure 6). ORP5 and ORP8 are lipid exchangers, each of which contains an oxysterol-related domain (ORD) [73,74,75,76]. The hydrophobic cavity within the ORDs of ORP5 and ORP8 specifically bind with PS and PI4P with high affinity [73,74,75,76]. Thus, ORP5 and ORP8 actively exchange PI4P in the PM inner leaflet and PS in the ER [73,74,75,76]. EM-nanoclustering analysis and confocal imaging consistently showed that knocking down ORP5 and/or ORP8 effectively mislocalized the GFP-tagged PS probe, LactC2, on the PM inner leaflet, suggesting that ORP5/ORP8 knockdown reduced the PS levels in the PM [80]. In consequence, ORP5/ORP8 knockdown mislocalized GFP-KRASG12V from the PM, disrupted the nanoclustering of GFP-KRASG12V left on the PM [80]. Concordantly, ORP5/ORP8 knockdown effectively reduced the number of colonies of the mutant KRAS-dependent pancreatic cancer cells without affecting the cancer cells independent of mutant KRAS [80]. Consistent with the concept of PI4P/PS exchange for the enrichment of PS in the PM, pharmaceutically inhibiting a class III PI4P kinase, PI4PKIIIα, via a specific PI4PKIIIα inhibitor Compound 7 (C7), compromised the spatiotemporal organization of GFP-KRASG12V on the PM, and reduced the colony number of the mutant KRAS-dependent pancreatic tumor cells in colony counting assays [80]. Thus, interfering with the PS exchange between the PM and the ER effectively depletes PS contents in the PM, compromises the nanoclustering and function of oncogenic mutant KRAS and specifically attenuates the tumor activities of the KRAS-dependent tumor cells.

### 5.3. Perturbation of Lipid Sorting Specificity of KRAS Polybasic Domain

The polybasic domain structure of the oncogenic mutant KRAS can also be manipulated pharmacologically to alter the lipid preferences of KRAS (Figure 6). Phosphorylation of Serine 181 adds a negatively charged phosphate group immediately adjacent to a polybasic domain (Lys 175–180) alters the electrostatic interactions between the KRAS polybasic domain and the anionic surface of the PM inner leaflet. Indeed, all-atom MD simulations predicted that the phosphorylated KRAS polybasic domain backbone underwent distinct conformational sampling than the unphosphorylated KRAS [23]. While ~64% of the unphosphorylated polybasic domain sampled the D states and the remaining 36% sampled ordered O and I states, the phosphorylated KRAS adopted exclusively the disordered D states [23]. This shift in conformational sampling resulted in a weakened hydrogen bonding between individual lysine residues within the phosphorylated polybasic domain and PS and PC headgroups [23]. In consequence, the phosphorylated GFP-KRASG12V no longer associated with PS, determined in parallel EM-bivariate co-clustering analysis [23]. Rather, the RFP-tagged phosphorylated KRAS co-clustered extensively with GFP-PH-PLCδ (a specific PIP2-binding domain) and GFP-PH-Akt (a specific PIP3-binding domain) [23], suggesting that the phosphorylated KRAS shifts its lipid preference from the highly abundant PS to minor lipid types PIP2 and PIP3. Since PIP2 and PIP3 are minor lipid types in the PM (<1% of the total lipid contents in the PM inner leaflet), the association of the phosphorylated KRAS with the PM is weakened. Serine 181 of KRAS can be phosphorylated by protein kinase C (PKC) and protein kinase G (PKG) [81,82]. Indeed, treatment of bryostatin-1, a potent activator of PKC, effectively phosphorylated Serine 181 of KRAS, mislocalized an oncogenic mutant KRAS from the PM and promoted apoptosis of the mutant KRAS-dependent tumor cells [81]. Small molecule agonists of the PKG pathway also efficiently phosphorylated Serine 181 of KRAS, which in turn mislocalized KRAS from the PM, disrupted the nanoclustering of the phosphorylated KRAS and compromised the KRAS-dependent MAPK signaling [82]. The PKG cascade includes AMP-activated protein kinase (AMPK), eNOS, soluble guanylyl cyclase (sGC), cyclic GMP (cGMP) and PKG. Various components in the PKG pathway can be pharmacologically targeted to elevate the accumulation of cGMP, in turn phosphorylate Serine 181 of oncogenic mutant KRAS, compromise the nanoclustering and signaling of mutant KRAS. These small molecule targeting the PKG pathway include AMPK activators, such as oligomycin A, neoantimycin, antidiabetic drug metformin and aminoimidazole-4-carboxamide riboside (AICAR), nitric oxide donor (diethylamine nitric oxide, DEA-NO), an inhibitor of phosphodiesterase 5 (Sildenafil) [82]. Thus, pharmacologically inducing phosphorylation of mutant KRAS interfers with the spatiotemporal organization and signaling of mutant KRAS.

## 6. Conclusions

RAS small GTPases, especially KRAS, have been difficult drug targets. Alternatively, the plasma membrane (PM) is the main signaling compartment for RAS small GTPases, thus a promising target. However, initial attempts of using farnesyltransferase inhibitors to mislocalize KRAS from the PM have not been successful. This has dampened the enthusiasm of targeting the KRAS membrane interactions. Recent advances in super-resolution imaging methodology have allowed further characterization of the spatiotemporal organization of RAS proteins, especially KRAS, on the PM. In the current review, we discussed recent advances in our understanding of the intriguing capabilities of KRAS to selectively sort not only lipid headgroups but also lipid acyl chains. These specificities reignite the possibility of targeting KRAS/membrane interactions to interfere with its oncogenesis. Because of the distinct roles of PS lipids, specifically the mixed-chain PS species, in the signaling nanoclusters of KRAS, the precisely regulated metabolism and transport of PS lipids can be targeted to impact KRAS oncogenesis. The intriguing capability of KRAS to selectively sort mixed-chain PS species can also be targeted to disrupt the structural integrity of KRAS nanoclusters, and in turn to compromise KRAS oncogenesis. Important questions still remain, such as how KRAS uses different features of its C-terminal membrane-anchoring domain, as well as its G-domain, to selectively sort different lipids. Does dimerization of KRAS contribute and/or depend on distinct lipids? How do other protein components within KRAS nanoclusters, such as galectin 3, contribute to the distinct lipid enrichment in these nano-domains? These aspects will provide further specificities of lipid sorting, thus making targeting these elements more easily.

## Data Availability

Not applicable.

## References

[1] Cox A.D., Der C.J., Philips M.R. (2015). Targeting RAS Membrane Association: Back to the Future for Anti-RAS Drug Discovery?. Clin. Cancer Res..

[2] Cox A.D., Fesik S.W., Kimmelman A.C., Luo J., Der C.J. (2014). Drugging the undruggable RAS: Mission possible?. Nat. Rev. Drug Discov..

[3] Downward J. (2003). Targeting RAS signalling pathways in cancer therapy. Nat. Rev. Cancer.

[4] Hancock J.F. (2003). Ras proteins: Different signals from different locations. Nat. Rev. Mol. Cell Biol..

[5] Papke B., Der C.J. (2017). Drugging RAS: Know the enemy. Science.

[6] Prior I.A., Hood F.E., Hartley J.L. (2020). The Frequency of Ras Mutations in Cancer. Cancer Res..

[7] Veatch S.L. (2008). Lipids out of order. Nat. Chem. Biol..

[8] Veatch S.L., Keller S.L. (2002). Organization in lipid membranes containing cholesterol. Phys. Rev. Lett..

[9] Simons K., Gerl M.J. (2010). Revitalizing membrane rafts: New tools and insights. Nat. Rev. Mol. Cell Biol..

[10] Baumgart T., Hess S.T., Webb W.W. (2003). Imaging coexisting fluid domains in biomembrane models coupling curvature and line tension. Nature.

[11] Prior I.A., Muncke C., Parton R.G., Hancock J.F. (2003). Direct visualization of Ras proteins in spatially distinct cell surface microdomains. J. Cell Biol..

[12] Plowman S.J., Muncke C., Parton R.G., Hancock J.F. (2005). H-ras, K-ras, and inner plasma membrane raft proteins operate in nanoclusters with differential dependence on the actin cytoskeleton. Proc. Natl. Acad. Sci. USA.

[13] Nicolini C., Baranski J., Schlummer S., Palomo J., Lumbierres-Burgues M., Kahms M., Kuhlmann J., Sanchez S., Gratton E., Waldmann H. (2006). Visualizing Association of N-Ras in Lipid Microdomains: Influence of Domain Structure and Interfacial Adsorption. J. Am. Chem. Soc..

[14] Tian T., Harding A., Inder K., Plowman S., Parton R.G., Hancock J.F. (2007). Plasma membrane nanoswitches generate high-fidelity Ras signal transduction. Nat. Cell Biol..

[15] Weise K., Triola G., Brunsveld L., Waldmann H., Winter R. (2009). Influence of the lipidation motif on the partitioning and association of N-Ras in model membrane subdomains. J. Am. Chem. Soc..

[16] Weise K., Kapoor S., Denter C., Nikolaus J., Opitz N., Koch S., Triola G., Herrmann A., Waldmann H., Winter R. (2011). Membrane-mediated induction and sorting of K-Ras microdomain signaling platforms. J. Am. Chem. Soc..

[17] Janosi L., Li Z., Hancock J.F., Gorfe A.A. (2012). Organization, dynamics, and segregation of Ras nanoclusters in membrane domains. Proc. Natl. Acad. Sci. USA.

[18] Kapoor S., Triola G., Vetter I.R., Erlkamp M., Waldmann H., Winter R. (2012). Revealing conformational substates of lipidated N-Ras protein by pressure modulation. Proc. Natl. Acad. Sci. USA.

[19] Li Z., Janosi L., Gorfe A.A. (2012). Formation and domain partitioning of H-ras peptide nanoclusters: Effects of peptide concentration and lipid composition. J. Am. Chem. Soc..

[20] Kapoor S., Weise K., Erlkamp M., Triola G., Waldmann H., Winter R. (2012). The role of G-domain orientation and nucleotide state on the Ras isoform-specific membrane interaction. Eur. Biophys. J..

[21] Zhou Y., Liang H., Rodkey T., Ariotti N., Parton R.G., Hancock J.F. (2014). Signal Integration by Lipid-Mediated Spatial Cross Talk between Ras Nanoclusters. Mol. Cell Biol..

[22] Zhou Y., Wong C.O., Cho K.J., van der Hoeven D., Liang H., Thakur D.P., Luo J., Babic M., Zinsmaier K.E., Zhu M.X. (2015). SIGNAL TRANSDUCTION. Membrane potential modulates plasma membrane phospholipid dynamics and K-Ras signaling. Science.

[23] Zhou Y., Prakash P., Liang H., Cho K.J., Gorfe A.A., Hancock J.F. (2017). Lipid-Sorting Specificity Encoded in K-Ras Membrane Anchor Regulates Signal Output. Cell.

[24] Liang H., Mu H., Jean-Francois F., Lakshman B., Sarkar-Banerjee S., Zhuang Y., Zeng Y., Gao W., Zaske A.M., Nissley D.V. (2019). Membrane curvature sensing of the lipid-anchored K-Ras small GTPase. Life Sci. Alliance.

[25] Zhou Y., Prakash P.S., Liang H., Gorfe A.A., Hancock J.F. (2021). The KRAS and other prenylated polybasic domain membrane anchors recognize phosphatidylserine acyl chain structure. Proc. Natl. Acad. Sci. USA.

[26] Zhou Y., Ariotti N., Rae J., Liang H., Tillu V., Tee S., Bastiani M., Bademosi A.T., Collins B.M., Meunier F.A. (2021). Caveolin-1 and cavin1 act synergistically to generate a unique lipid environment in caveolae. J. Cell Biol..

[27] Murakoshi H., Iino R., Kobayashi T., Fujiwara T., Ohshima C., Yoshimura A., Kusumi A. (2004). Single-molecule imaging analysis of Ras activation in living cells. Proc. Natl. Acad. Sci. USA.

[28] Inder K., Hancock J.F. (2008). System output of the MAPK module is spatially regulated. Commun. Integr. Biol..

[29] Inder K., Harding A., Plowman S.J., Philips M.R., Parton R.G., Hancock J.F. (2008). Activation of the MAPK module from different spatial locations generates distinct system outputs. Mol. Biol. Cell..

[30] Belanis L., Plowman S.J., Rotblat B., Hancock J.F., Kloog Y. (2008). Galectin-1 is a novel structural component and a major regulator of h-ras nanoclusters. Mol. Biol. Cell.

[31] Blazevits O., Mideksa Y.G., Solman M., Ligabue A., Ariotti N., Nakhaeizadeh H., Fansa E.K., Papageorgiou A.C., Wittinghofer A., Ahmadian M.R. (2016). Galectin-1 dimers can scaffold Raf-effectors to increase H-ras nanoclustering. Sci. Rep..

[32] Posada I.M.D., Lectez B., Sharma M., Oetken-Lindholm C., Yetukuri L., Zhou Y., Aittokallio T., Abankwa D. (2017). Rapalogs can promote cancer cell stemness in vitro in a Galectin-1 and H-ras-dependent manner. Oncotarget.

[33] Rotblat B., Niv H., Andre S., Kaltner H., Gabius H.J., Kloog Y. (2004). Galectin-1(L11A) predicted from a computed galectin-1 farnesyl-binding pocket selectively inhibits Ras-GTP. Cancer Res..

[34] Elad-Sfadia G., Haklai R., Balan E., Kloog Y. (2004). Galectin-3 augments K-Ras activation and triggers a Ras signal that attenuates ERK but not phosphoinositide 3-kinase activity. J. Biol. Chem..

[35] Shalom-Feuerstein R., Plowman S.J., Rotblat B., Ariotti N., Tian T., Hancock J.F., Kloog Y. (2008). K-ras nanoclustering is subverted by overexpression of the scaffold protein galectin-3. Cancer Res..

[36] Posada I.M., Serulla M., Zhou Y., Oetken-Lindholm C., Abankwa D., Lectez B. (2016). ASPP2 Is a Novel Pan-Ras Nanocluster Scaffold. PLoS ONE.

[37] Inder K.L., Hill M.M., Hancock J.F. (2010). Nucleophosmin and nucleolin regulate K-Ras signaling. Commun. Integr. Biol..

[38] Inder K.L., Lau C., Loo D., Chaudhary N., Goodall A., Martin S., Jones A., van der Hoeven D., Parton R.G., Hill M.M. (2009). Nucleophosmin and nucleolin regulate K-Ras plasma membrane interactions and MAPK signal transduction. J. Biol. Chem..

[39] Zhou Y., Hancock J.F. (2015). Ras nanoclusters: Versatile lipid-based signaling platforms. Biochim. Biophys. Acta.

[40] Zhou Y., Hancock J.F. (2018). Deciphering lipid codes: K-Ras as a paradigm. Traffic.

[41] Zhou Y., Hancock J.F. (2020). A novel prenyl-polybasic domain code determines lipid-binding specificity of the K-Ras membrane anchor. Small GTPases.

[42] Ghosh S., Strum J.C., Sciorra V.A., Daniel L., Bell R.M. (1996). Raf-1 kinase possesses distinct binding domains for phosphatidylserine and phosphatidic acid. Phosphatidic acid regulates the translocation of Raf-1 in 12-O-tetradecanoylphorbol-13-acetate-stimulated Madin-Darby canine kidney cells. J. Biol. Chem..

[43] Ghosh S., Xie W.Q., Quest A.F., Mabrouk G.M., Strum J.C., Bell R.M. (1994). The cysteine-rich region of raf-1 kinase contains zinc, translocates to liposomes, and is adjacent to a segment that binds GTP-ras. J. Biol. Chem..

[44] Li Z.L., Prakash P., Buck M. (2018). A “Tug of War” Maintains a Dynamic Protein-Membrane Complex: Molecular Dynamics Simulations of C-Raf RBD-CRD Bound to K-Ras4B at an Anionic Membrane. ACS Cent. Sci..

[45] Packer M.R., Parker J.A., Chung J.K., Li Z., Lee Y.K., Cookis T., Guterres H., Alvarez S., Hossain M.A., Donnelly D.P. (2021). Raf promotes dimerization of the Ras G-domain with increased allosteric connections. Proc. Natl. Acad. Sci. USA.

[46] Castellano E., Downward J. (2011). RAS Interaction with PI3K: More Than Just Another Effector Pathway. Genes Cancer.

[47] Hemmings B.A., Restuccia D.F. (2012). PI3K-PKB/Akt pathway. Cold Spring Harb. Perspect. Biol..

[48] Miao B., Skidan I., Yang J., Lugovskoy A., Reibarkh M., Long K., Brazell T., Durugkar K.A., Maki J., Ramana C.V. (2010). Small molecule inhibition of phosphatidylinositol-3,4,5-triphosphate (PIP3) binding to pleckstrin homology domains. Proc. Natl. Acad. Sci. USA.

[49] Zhou Y., Hancock J.F. (2021). Super-Resolution Imaging and Spatial Analysis of RAS on Intact Plasma Membrane Sheets. Methods Mol. Biol..

[50] Zhou Y., Hancock J.F. (2018). Electron microscopy combined with spatial analysis: Quantitative mapping of the nano-assemblies of plasma membrane-associating proteins and lipids. Biophys. Rep..

[51] Sarkar-Banerjee S., Sayyed-Ahmad A., Prakash P., Cho K.J., Waxham M.N., Hancock J.F., Gorfe A.A. (2017). Spatiotemporal Analysis of K-Ras Plasma Membrane Interactions Reveals Multiple High Order Homo-oligomeric Complexes. J. Am. Chem. Soc..

[52] Hancock J.F., Paterson H., Marshall C.J. (1990). A polybasic domain or palmitoylation is required in addition to the CAAX motif to localize p21ras to the plasma membrane. Cell.

[53] Hancock J.F., Cadwallader K., Paterson H., Marshall C.J. (1991). A CAAX or a CAAL motif and a second signal are sufficient for plasma membrane targeting of ras proteins. EMBO J..

[54] Lee S., Uchida Y., Emoto K., Umeda M., Kuge O., Taguchi T., Arai H. (2012). Impaired retrograde membrane traffic through endosomes in a mutant CHO cell defective in phosphatidylserine synthesis. Genes Cells.

[55] Cho K.J., van der Hoeven D., Zhou Y., Maekawa M., Ma X., Chen W., Fairn G.D., Hancock J.F. (2015). Inhibition of Acid Sphingomyelinase Depletes Cellular Phosphatidylserine and Mislocalizes K-Ras from the Plasma Membrane. Mol. Cell Biol..

[56] Cho K.J., Park J.H., Piggott A.M., Salim A.A., Gorfe A.A., Parton R.G., Capon R.J., Lacey E., Hancock J.F. (2012). Staurosporines disrupt phosphatidylserine trafficking and mislocalize Ras proteins. J. Biol. Chem..

[57] van der Hoeven D., Cho K.J., Ma X., Chigurupati S., Parton R.G., Hancock J.F. (2013). Fendiline inhibits K-Ras plasma membrane localization and blocks K-Ras signal transmission. Mol. Cell Biol..

[58] van der Hoeven D., Cho K.J., Zhou Y., Ma X., Chen W., Naji A., Montufar-Solis D., Zuo Y., Kovar S.E., Levental K.R. (2017). Sphingomyelin metabolism is a regulator of KRAS function. Mol. Cell Biol..

[59] Lakshman B., Messing S., Schmid E.M., Clogston J.D., Gillette W.K., Esposito D., Kessing B., Fletcher D.A., Nissley D.V., McCormick F. (2019). Quantitative biophysical analysis defines key components modulating recruitment of the GTPase KRAS to the plasma membrane. J. Biol. Chem..

[60] Janosi L., Gorfe A.A. (2010). Segregation of negatively charged phospholipids by the polycationic and farnesylated membrane anchor of Kras. Biophys. J..

[61] Rawicz W., Olbrich K.C., McIntosh T., Needham D., Evans E. (2000). Effect of chain length and unsaturation on elasticity of lipid bilayers. Biophys. J..

[62] Blackiston D.J., McLaughlin K.A., Levin M. (2009). Bioelectric controls of cell proliferation: Ion channels, membrane voltage and the cell cycle. Cell Cycle.

[63] Lang F., Foller M., Lang K.S., Lang P.A., Ritter M., Gulbins E., Vereninov A., Huber S.M. (2005). Ion channels in cell proliferation and apoptotic cell death. J. Membr. Biol..

[64] Pardo L.A. (2004). Voltage-gated potassium channels in cell proliferation. Physiology.

[65] Szabo I., Zoratti M., Gulbins E. (2010). Contribution of voltage-gated potassium channels to the regulation of apoptosis. FEBS Lett..

[66] Miroshnikova Y.A., Le H.Q., Schneider D., Thalheim T., Rubsam M., Bremicker N., Polleux J., Kamprad N., Tarantola M., Wang I. (2018). Adhesion forces and cortical tension couple cell proliferation and differentiation to drive epidermal stratification. Nat. Cell Biol..

[67] Hirama T., Lu S.M., Kay J.G., Maekawa M., Kozlov M.M., Grinstein S., Fairn G.D. (2017). Membrane curvature induced by proximity of anionic phospholipids can initiate endocytosis. Nat. Commun..

[68] Thakar R.G., Cheng Q., Patel S., Chu J., Nasir M., Liepmann D., Komvopoulos K., Li S. (2009). Cell-shape regulation of smooth muscle cell proliferation. Biophys. J..

[69] Roy S., Plowman S., Rotblat B., Prior I.A., Muncke C., Grainger S., Parton R.G., Henis Y.I., Kloog Y., Hancock J.F. (2005). Individual palmitoyl residues serve distinct roles in H-ras trafficking, microlocalization, and signaling. Mol. Cell Biol..

[70] Schmick M., Vartak N., Papke B., Kovacevic M., Truxius D.C., Rossmannek L., Bastiaens P.I. (2014). KRas localizes to the plasma membrane by spatial cycles of solubilization, trapping and vesicular transport. Cell.

[71] Zimmermann G., Papke B., Ismail S., Vartak N., Chandra A., Hoffmann M., Hahn S.A., Triola G., Wittinghofer A., Bastiaens P.I. (2013). Small molecule inhibition of the KRAS-PDEdelta interaction impairs oncogenic KRAS signalling. Nature.

[72] Chandra A., Grecco H.E., Pisupati V., Perera D., Cassidy L., Skoulidis F., Ismail S.A., Hedberg C., Hanzal-Bayer M., Venkitaraman A.R. (2011). The GDI-like solubilizing factor PDEdelta sustains the spatial organization and signalling of Ras family proteins. Nat. Cell Biol..

[73] Moser von Filseck J., Copic A., Delfosse V., Vanni S., Jackson C.L., Bourguet W., Drin G. (2015). INTRACELLULAR TRANSPORT. Phosphatidylserine transport by ORP/Osh proteins is driven by phosphatidylinositol 4-phosphate. Science.

[74] Moser von Filseck J., Vanni S., Mesmin B., Antonny B., Drin G. (2015). A phosphatidylinositol-4-phosphate powered exchange mechanism to create a lipid gradient between membranes. Nat. Commun..

[75] Galmes R., Houcine A., van Vliet A.R., Agostinis P., Jackson C.L., Giordano F. (2016). ORP5/ORP8 localize to endoplasmic reticulum-mitochondria contacts and are involved in mitochondrial function. EMBO Rep..

[76] Sohn M., Ivanova P., Brown H.A., Toth D.J., Varnai P., Kim Y.J., Balla T. (2016). Lenz-Majewski mutations in PTDSS1 affect phosphatidylinositol 4-phosphate metabolism at ER-PM and ER-Golgi junctions. Proc. Natl. Acad. Sci. USA.

[77] Gulbins E., Palmada M., Reichel M., Luth A., Bohmer C., Amato D., Muller C.P., Tischbirek C.H., Groemer T.W., Tabatabai G. (2013). Acid sphingomyelinase-ceramide system mediates effects of antidepressant drugs. Nat. Med..

[78] Muhle C., Huttner H.B., Walter S., Reichel M., Canneva F., Lewczuk P., Gulbins E., Kornhuber J. (2013). Characterization of acid sphingomyelinase activity in human cerebrospinal fluid. PLoS ONE.

[79] Munzer P., Borst O., Walker B., Schmid E., Feijge M.A., Cosemans J.M., Chatterjee M., Schmidt E.M., Schmidt S., Towhid S.T. (2014). Acid sphingomyelinase regulates platelet cell membrane scrambling, secretion, and thrombus formation. Arterioscler. Thromb. Vasc. Biol..

[80] Kattan W.E., Chen W., Ma X., Lan T.H., van der Hoeven D., van der Hoeven R., Hancock J.F. (2019). Targeting plasma membrane phosphatidylserine content to inhibit oncogenic KRAS function. Life Sci. Alliance.

[81] Bivona T.G., Quatela S.E., Bodemann B.O., Ahearn I.M., Soskis M.J., Mor A., Miura J., Wiener H.H., Wright L., Saba S.G. (2006). PKC regulates a farnesyl-electrostatic switch on K-Ras that promotes its association with Bcl-XL on mitochondria and induces apoptosis. Mol. Cell.

[82] Cho K.J., Casteel D.E., Prakash P., Tan L., van der Hoeven D., Salim A.A., Kim C., Capon R.J., Lacey E., Cunha S.R. (2016). AMPK and Endothelial Nitric Oxide Synthase Signaling Regulates K-Ras Plasma Membrane Interactions via Cyclic GMP-Dependent Protein Kinase 2. Mol. Cell Biol..

